# Caregiving in the COVID‐19 pandemic: Family adaptations following an intensive care unit hospitalisation

**DOI:** 10.1111/jocn.16560

**Published:** 2022-10-19

**Authors:** Sheria G. Robinson‐Lane, Amanda N. Leggett, Florence U. Johnson, Natalie Leonard, Alicia G. Carmichael, Grace Oxford, Tanbirul Miah, Johnny J. Wright, Amanda C. Blok, Theodore J. Iwashyna, Richard Gonzalez

**Affiliations:** ^1^ Department of Systems, Populations, and Leadership University of Michigan School of Nursing Ann Arbor Michigan USA; ^2^ Department of Psychiatry University of Michigan Medical School Ann Arbor Michigan USA; ^3^ BioSocial Methods Collaborative, Research Center for Group Dynamics University of Michigan Institute for Social Research Ann Arbor Michigan USA; ^4^ VA Center for Clinical Management Research, VA Ann Arbor Healthcare System Ann Arbor Michigan USA

**Keywords:** caregiver, coronavirus, family, follow‐up studies, inpatients, psychological adaptation, qualitative research

## Abstract

**Aim and Objective:**

To identify how family caregivers adapt to the caregiving role following a relative's COVID‐19‐related intensive care unit (ICU) hospitalisation.

**Background:**

Family caregiving is often associated with poor health amongst caregivers which may limit their capacity to effectively support patients. Though severe COVID‐19 infection has necessitated increasing numbers of persons who require caregiver support, little is known about these caregivers, the persons they are caring for, or the strategies used to effectively adjust to the caregiving role.

**Design:**

A qualitative descriptive study design was adopted, and findings are reported using COREQ.

**Methods:**

A secondary analysis of transcripts from semi‐structured interviews conducted with recently discharged ICU patients who had COVID‐19 (*n* = 16) and their family caregivers (*n* = 16) was completed using thematic analysis. MAXQDA 2020 and Miro were used to organise data and complete coding. Analysis involved a structured process of open and closed coding to identify and confirm themes that elucidated adaptation to family caregiving.

**Results:**

Six themes highlight how family caregivers adapt to the caregiving role following an ICU COVID‐19‐related hospitalisation including (1) engaging the support of family and friends, (2) increased responsibilities to accommodate caregiving, (3) managing emotions, (4) managing infection control, (5) addressing patient independence and (6) engaging support services. These themes were found to be congruent with the Roy adaptation model.

**Conclusions:**

Family caregiving is a stressful transition following a patient's acute hospitalisation. Effective adaptation requires flexibility and sufficient support, beginning with the care team who can adequately prepare the family for the anticipated challenges of recovery.

**Relevance to Clinical Practice:**

Clinical teams may improve post‐hospitalisation care outcomes of patients by preparing families to effectively adjust to the caregiver role—particularly in identifying sufficient support resources.

**Patient or Public Contribution:**

Participation of patients/caregivers in this study was limited to the data provided through participant interviews.

**What does this paper contribute to the wider global community?**

- This study provides insights into the strategies that nurses may use to improve post‐discharge outcomes for families following a COVID‐19‐related ICU hospitalisation.
- Family‐centred discharge planning is highlighted as an important approach that ensures both patient and caregiver needs are met.
- Adequate social support is identified as a key factor in effective adaptation to family caregiving.

## 1 INTRODUCTION

Family caregiving, or providing unpaid medical, behavioural, or other care related to an illness or disability, has long been the mechanism by which families and friends are able to support the health‐related needs of loved ones outside of institutional care environments such as hospitals and nursing homes (Jack‐Ide et al., 2013; Mendez‐Luck et al., 2009; Schulz et al., 2020). In fact, Florence Nightingale's famed book *Notes on Nursing* (1924) was developed with the aim of offering “hints” on how to provide sufficient home nursing care should the occasion arise when one found oneself in “charge of somebody's health” (p. 1). The emergence of the severe acute respiratory syndrome coronavirus (SARS‐CoV‐2), and subsequently more than 609 million coronavirus disease 2019 (COVID‐19) infections around the world (World Health Organization [WHO], 2022), has surely led to many individuals being responsible for the health of another.

Approximately 21% of COVID‐19 hospitalised patients require care in an intensive care unit (ICU) (Centers for Disease Control and Prevention [CDC], 2022) for respiratory and/or cardiovascular complications. Following a COVID‐19 ICU hospitalisation, patients often experience common symptoms such as ongoing cough, shortness of breath, generalised weakness, fatigue, chest pain, memory deficits, confusion, and psychological distress that may take weeks to years to recover from—if at all (Halpin et al., 2020; Ogoina et al., 2021; Parotto et al., 2021). Further, patients who have been discharged from the ICU with health conditions similar to COVID‐19, such as acute respiratory distress syndrome, often require the support of family members to assist in disability management, recovery encouragement, and identification of formal support services (Hauschildt et al., 2021). While the support of family and friends following a severe COVID‐19 infection requiring ICU hospitalisation is clearly necessary, there is a dearth of literature about the post‐hospitalisation COVID‐19 family caregiving experience, particularly information about the families, the patients they are assisting, or how clinicians may best support families in adjusting to the caregiving role. Examining the dyadic experiences of recently discharged ICU patients who have had COVID‐19 and their family caregivers may allow for a deeper understanding of these experiences and lead to improved family health outcomes.

## 2 BACKGROUND

Family caregiving has long been recognised as a formal role with economic, social, physical and psychological consequences for care providers including financial loss, social isolation, loneliness, depression, anxiety and worsened overall health (AARP & National Alliance for Caregiving, 2020; Capistrant, 2016; Grice, 2020). Similar findings have been noted in the few studies examining family caregivers of patients with COVID‐19, particularly, financial challenges, stress, anxiety, insomnia, and social isolation (Picardi et al., 2021; Rahimi et al., 2021). It is common for caregivers, across patient diagnoses, to become unexpectedly engaged in family caregiving responsibilities, with little to no training, following a patient's acute illness, or medical event, and subsequent hospitalisation (AARP & National Alliance for Caregiving, 2020; Naylor et al., 2017; Slatyer et al., 2019). Presently, the COVID‐19 pandemic has been the primary source of acute hospitalisations amongst older adults, with most surviving adults who were admitted from home, returning home following hospital discharge (CDC, 2021a, 2021b; Chopra et al., 2020; Robinson‐Lane, Sutton, et al., 2021). Though families have long reported dissatisfaction with the hospital discharge process (Bauer et al., 2009), legislation has been passed, or will soon pass, in most U.S. states that require the better engagement of family caregivers in the hospital discharge planning process, including providing any necessary education or training (AARP, 2014; Administration for Community Living [ACL], 2021). Despite these efforts, pandemic‐initiated policies aimed at reducing infection spread within hospitals have kept caregivers away and perhaps inadvertently further reduced caregiver preparedness for patient discharge (Lloreda, 2021).

Underprepared caregivers are more likely to experience increased insomnia, stress, depression and anxiety; symptoms that not only directly affect caregiver health but can also influence the perception of care burden, the familial relationship quality, and both mental and cognitive health outcomes in patients (Picardi et al., 2021; Robinson‐Lane, Zhang, & Patel, 2021; Snyder et al., 2015; Tschanz et al., 2013). Of particular concern, poor mental health outcomes amongst family caregivers have been related to poor physical health and diminished ability to fulfil caregiving roles, increasing the likelihood of alternative placements for patients such as assisted living communities or nursing homes (Chang et al., 2010; Schulz & Sherwood, 2008). In contrast, caregivers who have been more actively engaged in discharge planning felt better prepared, had higher satisfaction and acceptance of the caregiving role, and reported better health than caregivers who were less engaged (Bull et al., 2000). Recent study findings suggest that caregiving during the COVID‐19 pandemic has resulted in even more psychological distress for family caregivers, caregiver burden, and loneliness than pre‐pandemic (Archer et al., 2021; Beach et al., 2021; Leggett et al., 2021), thus highlighting a critical need for sufficient family caregiver support.

As a mass disabling event, the COVID‐19 pandemic will continue to result in challenges to patients' physical, cognitive, and mental health that will likely require the ongoing support of their families (Crook et al., 2021; Mandal et al., 2021; Roth et al., 2021). Examining how the family, particularly the patient/caregiver dyad, adjusts to manage COVID‐19‐related disability and related caregiving needs following an ICU hospitalisation can provide important insights about family support requirements and critical intervention points both pre‐ and post‐hospital discharge. To this end, the following examines the dyadic post‐discharge experiences of family caregivers and their relatives who were hospitalised in an ICU with COVID‐19‐related infection.

## 3 METHODS

### 3.1 Design

Qualitative descriptive research methods facilitate the inductive discovery of patterns and themes around a life event (Parse, 2001; Powers & Knapp, 2010; Vaismoradi et al., 2013). As such, a qualitative descriptive study design was employed to answer the research question, “How does one adapt to family caregiving following an ICU COVID‐19‐related hospitalization?” The Consolidated Criteria for Reporting Qualitative Research (COREQ) (Tong et al., 2007) was used to report the findings from this study (See Supporting Information File S1).

### 3.2 Data and study participants

Data from the Health Enhanced by Adjusting and Recovering Together (HEART) COVID‐19 Recovery Project (Research Center for Group Dynamics, 2021) was used for this secondary data analysis. The HEART study recruited dyads of family caregivers and patients hospitalised in the U.S. with COVID‐19 at the start of the U.S. pandemic through October 2020 in Southeastern Michigan. The study explored patient and caregiver COVID‐19 experiences from initial illness through hospitalisation and recovery at home, with the aim of identifying areas for rapid intervention. The primary product of this work was a patient and family collaborative care management guide (BioSocial Methods Collaborative, 2020). Eligible study participants were English‐speaking dyads at least 18 years old in which one dyad member was the family caregiver of a recently discharged patient and the patient met the following inclusion criteria (1) hospitalised as a result of a positive COVID‐19 test, (2) spent at least 3 days in an inpatient unit during hospitalisation, (3) experienced heated high‐flow nasal cannula, non‐invasive positive pressure ventilation or mechanical ventilation of Fi02 > 60% during their hospital stay (as noted in the medical record) and (4) discharged to home from the hospital. Dyads were recruited through multiple methods including: (1) social media campaigns (Facebook, Instagram), (2) a study website, (3) a research volunteer web portal and (4) targeted letters, emails and phone calls using a patient database.

### 3.3 Data collection

Semi‐structured interviews were conducted with each dyad member (*n* = 32), by a diverse team of male and female research staff trained in qualitative interviewing. Each interview took an average of 59.2 minutes (see Table S1). Co‐authors (ANL, AGC, GO) participated in interviewing participants and had no prior connection to, or relationship with, participants. Interview questions were developed by a team of clinicians and health service scientists. Interviews focused on patient and caregiver experiences in providing and receiving care for a severe COVID‐19 infection and the immediate recovery period following hospitalisation. Interviews were conducted remotely via phone or video conferencing based on participant preference and internet connection availability. All interviews were audio‐recorded and verbatim transcribed. As dyads were self‐selected to participate in the study, which was limited to one‐time interviews of each caregiver and patient, and a brief electronic demographic survey, there was no attrition.

### 3.4 Data analysis

Braun and Clarke's (2006) approach, which supports the examination of dyadic interviews, was used to complete the analysis. MAXQDA 2020 (VERBI Software, 2019) and Miro (2020) were used to organise data and complete coding. A team of four coders individually completed the initial open coding of all transcripts (*n* = 32, 16 caregiver transcripts, and 16 patient transcripts) with attention to the phenomena of adaptation to family caregiving. The research team then collaboratively reviewed and sorted all the codes and identified 14 themes. These themes were used to develop a codebook and guide for subsequent analysis (see Table 1). Next, two teams consisting of two coders each completed closed coding by reviewing the transcripts for the presence of the identified themes. This additional level of analysis helped to ensure reliability. The 14 themes were then ranked using our analytic software by frequency of coding occurrence across transcripts. This allowed us to identify themes most representative of participant experiences. Final themes were then further defined, refined and evaluated for relevancy to the phenomena of adaptation to family caregiving and application to relevant nursing theory.

**TABLE 1:** Codes, related themes and frequency of use across participants

Code	Definition	Code frequency	
		Caregiver	Patient
Family Support **Theme:** Engaging the support of friends and family	Support from friends and/or family members, neighbours, religious organisations, or other organisations or community members includes (but is not limited to) getting groceries, delivering medical supplies, providing transportation, and offering advice and emotional support that ultimately lessens the burden on the caregiver	16	16
Modifications **Theme:** Increased responsibilities	Big and small changes that are required to assist the care recipient with their physical health needs and overall care management. This may include changes in the caregiver's job status (hours worked or being able to work at all), alterations in everyday chores, or adjustments to the living space	16	15
Emotional Coping **Theme:** Managing emotions	The caregiver's expression of feelings (sadness, anxiety, worry, motivation, hope, etc.) about their responsibilities and the care recipient's health. Also, include what they are doing to deal with them. This can be a wide range of emotional coping strategies that the caregiver adopts to adjust to his/her new role and lifestyle	16	10
Precautions **Theme:** Attention to infection control	Changes in behaviour to prevent catching or spreading COVID‐19	15	15
Independence **Theme:** Addressing patient independence	A care recipient or caregiver asserting their ability to manage care needs on their own	12	12
Ancillary support **Theme:** Engaging Support Services	Support services provided by medical providers such as home care, therapy or monetary coverage ultimately lessen the burden of the caregiver	10	10
Wishes^a^	The expression of not having enough quality, quantity, or sufficient amount of assistance (such as financial, medical, emotional or social), information, or communication that will enable one to function or act optimally as a caregiver	12	14
DecisionMaker	The decision‐related roles and tasks that the caregiver adopts, due to the care recipient's temporary cognitive impairment caused by COVID‐19	14	9
CareComm	Caregiver's active role in advocating for care recipient's needs through communication and coordination with the health care team. Includes any communication between the caregiver and the health team regarding updates on the care recipient's status	10	6
Inside support	Any support that gives the caregiver an advantage by having direct or indirect access to someone inside the healthcare system who can provide information or access to resources, or contact with the care recipient, reduces the burden on the caregiver	8	7
Spiritual support	The degree to which a caregiver experiences or verbalises a connection to a higher power (i.e., God or other transcendent force) that the caregiver feels is actively supporting, protecting, guiding, helping, and healing	9	1
Stigma	A mark of disgrace or shame associated with those who have had or currently have COVID‐19. This leads to different treatment from others including shunning	6	6
Tech	Caregiver's adjustments to utilising computers, tablets, telephones, and other types of technology in different ways to care for the CR	3	1

^a^ Though there was a large frequency of occurrence, the wishes code was ultimately dropped from inclusion in the analysis as it was related to a specific interview question in which participants were asked what support they wished they had received. Based on the responses, we decided that there was ultimately little relevance to the adaptive experience of caregivers.

### 3.5 Ethical considerations

All eligible participants provided individual electronic written consent prior to study engagement and received a small stipend for their participation. Institutional Review Board approval was granted by (The University of Michigan (HUM00158390)) for both the primary study and this secondary analysis. All study team members completed training in human subjects research including the responsible conduct of research as required by the IRB. Study team composition included doctorally prepared psychologists, registered nurses and physicians, as well as graduate and undergraduate students, and non‐student/non‐medical personnel. No study team member had a prior relationship with any of the participants. Staff who conducted participant interviews also completed additional training on qualitative interview techniques. To ensure participant confidentiality, participants are identified as either a caregiver or patient along with a numerical identifier.

### 3.6 Methodological rigour

Credibility, transferability, dependability, and confirmability were established to ensure rigour in analysis (Lincoln & Guba, 1985). Triangulation, debriefing and review of collected data, along with referential adequacy, or comparison of preliminary findings against raw data, were used to ensure credibility. Investigator triangulation was ensured by using multiple independent coders. Consistency in initial data collection was ensured by using an interview script and reviewing recordings throughout the data collection period to provide feedback to interviewers as needed. In addition, inter‐rater reliability between coders was evaluated using kappa coefficient. If a kappa of less than 0.75 was noted, study team members met to review areas of discrepancy and reach consensus. The comprehensive reporting of the study sample, data collection methods, and interpretive process ensure transferability and confirmability, while our use of qualitative software tools and weekly team meetings helped to ensure dependability by allowing for process review, maintenance of an audit trail and team member reflexivity. Our results report the most significant and relevant themes illuminating the adaptive family caregiving experience.

## 4 RESULTS

The average age of caregivers in the sample was 54 (*SD* 13.3, range 18–74), and they provided care for patients with an average age of 58 (*SD* 13.2, range 28–78). While most caregivers were women (*n* = 12, 75%), most patients were men (*n* = 12, 75%). The sample was predominately White (*n* = 22, 68.8%) with the majority of caregivers and patients educated with at least some college (*n* = 28, 87.5%). Table 2 provides additional details on study participants.

**TABLE 2:** Demographic characteristics of patients (*n* = 16) and family caregivers (*n* = 16)

		Caregiver	Patient
Age (yrs; Mean [*SD*])		54 (13.3)	58 (13.2)
Sex (*n* [%])	Male	4 (25)	12 (75.0)
	Female	12 (75.0)	4 (25.0)
Race (*n* [%])	Black	4 (25.0)	4 (25.0)
	White	9 (56.2)	10 (62.5)
	Multi‐racial	1 (6.2)	1 (6.2)
Ethnicity (*n* [%])	Hispanic or Latinx	2 (12.5)	1 (6.2)
Relationship to patient (*n* [%])	Spouse or significant other	13 (81.3)	–
	Parent	1 (6.3)	–
	Adult Child	1 (6.3)	–
	Sibling	1 (6.3)	–
Education (*n* [%])	High School Diploma/GED	4 (25.0)	0 (0.0)
	Some College	4 (25.0)	8 (50.0)
	Bachelor's through advanced degrees	8 (50.0)	8 (50.0)
Income (*n* [%])	<$50,000	2 (12.5)	3 (18.8)
	$50,000–$74,999	2 (12.5)	3 (18.8)
	$75,000–$99,999	2 (12.5)	2 (12.5)
	≥$100,000	6 (37.5)	5 (31.2)
	Refused	4 (25.0)	3 (18.8)
Employment (*n* [%])	Employed full time	10 (62.5)	10 (62.5)
	Not employed	5 (31.2)	6 (37.5)
	Refused	1 (6.2)	0 (0.0)

Based upon patient/caregiver emphasis, six final themes were identified as key to the experience of adaptation to family caregiving following an ICU COVID‐19‐related hospitalisation. The themes included (1) engaging the support of family and friends, (2) increased responsibilities to accommodate caregiving, (3) managing emotions, (4) managing infection control, (5) addressing patient independence and (6) engaging support services. Each theme is supported by a participant quote with the participant number and role in the dyad noted in parentheses. The following explores each theme in more detail.

### 4.1 Themes

#### 4.1.1 Engaging the support of family and friends

Nobody was here but me [laughs] so I'd have my crying spell but I also had, like I said, family members, friends they would call and they would check on me 24/7. Sometimes it's like I'm on the phone all day then I'm on it again all night…but I appreciated everyone that took the time out to hear my mouth and listen to me. You know, and help me through it.(Caregiver 0174250)

The support of family and friends was a major theme expressed by the dyads that significantly contributed to the caregivers' ability to support their loved ones and maintain other responsibilities. This theme was derived from the code of *family support* and was used to indicate when friends, family members, neighbours, religious community members, and other communities that individuals of the dyad were a part of provided material assistance or emotional support that ultimately lessened the burden (physical or emotional) of the caregiver. This support included activities such as getting groceries, delivering medical supplies, providing transportation, offering advice, emotional support and prayers. As one caregiver noted, “You know, with family support and stuff, you know they're like there when you need them you know. You just say ‘hey’ and somebody's over doing something…even if it's just dropping [something] off on the porch” (Caregiver 0103405). Both patients and caregivers acknowledged the importance of these community connections to their overall well‐being. A patient noted, “There has been no shortness of support whatsoever from friends, family, to even strangers. So it's…yeah. The support has been great. And it's crucial, and I mean it's absolutely crucial” (Patient 1226314).

#### 4.1.2 Increased responsibilities to accommodate caregiving

You know, when I took him to the hospital, he was a very active, 210‐pound man. And bringing him home, he was in a wheelchair and 40 pounds lighter and unable to…he could walk very short distances but, needed one hundred percent of my help at all times, which I'm very grateful I have the ability to help him… We also have a business, and so I was trying to juggle, you know…Trying to keep our business floating, um there was a lot that crossed over…I guess, my biggest struggle was keeping everything flowing which I had to—and I did.(Caregiver 0106530).

A major transition for caregivers was taking on new responsibilities due to the inability of the patient to provide self‐care and manage tasks as they had before. These new responsibilities often lasted significantly longer than either the caregiver or their loved one anticipated and interfered with their ability to work. For example, while one caregiver described having to adjust their workday to come home more often to check on their loved one (Caregiver 0152964), another described having to take off work for 4 weeks to provide care (Caregiver 0101572). Particularly challenging to both caregivers and patients was the need for assistance with personal care needs. In addition to having little or no training to prepare them for their new role, caregivers found the tasks of caregiving to be both physically and emotionally exhausting. As one participant noted,

You know, when you have your spouse helping you and all of a sudden cannot do much, and he depends on 100 percent—it takes a toll on your body. You know, you are not used to doing all of this, then all of a sudden you have to do it. With a smile. Even though you are in pain or you are not sleeping well or whatever, does not matter. You have to hide it.(Caregiver 0144604)

This theme was derived from the code *modifications* and was applied when either the caregiver or patient noted an adjustment that the caregiver made to their daily routines, chores, employment or living space in order to assist the patient with their physical health needs or overall care management.

#### 4.1.3 Managing emotions

So when she came out of the hospital, she was like really aggressive, she felt like we were not giving her the level of care that she needed, even though like I was like right in the other room…I was trying to be as empathetic as possible and making myself available to her. And just trying to you know, take care as best as I could. You know crying and screaming after some nights because it was just like I did not understand—I thought she come out and be happy to be alive and she came out and like it was like whoa.(Caregiver 0154533)

The adjustment to the caregiving role, and particularly caring for a person at home following a precarious ICU hospitalisation, was a highly emotional experience. Caregivers described experiencing a large range of emotions including anxiety, fear, grief, emotional exhaustion and sadness—negative emotions that were often juxtaposed against the relief and joy they had that their loved one was home. As one participant aptly noted, “…emotionally it was up and down, you know. I did tell [my loved one] I didn't go through [having COVID] like you, but I went through it with you” (Caregiver 0174250). Caregivers found ways to effectively manage their negative emotions and continue delivering care. Most often, caregivers found support in working through their emotions by talking with family and friends. As another caregiver noted, “I never realized how much I needed to have those people present…” (Caregiver 0106530). This theme was derived from the *emotional coping* code and was used when the caregiver expressed any feelings about caregiving responsibilities, the patient's health, and/or anything that was done to manage emotions.

In addition to identifying major themes around the experience of adapting to the caregiving role following an acute hospitalisation, three additional themes were identified that appear to be unique to a COVID‐19‐related hospitalisation and the subsequent adjustment period.

#### 4.1.4 Managing infection control

You've had to keep family and friends away. I mean, I've had, like, a couple of my sisters say, “Hey, you want [me] to come and stay?”, and, you know, “You guys [should] get a break.” But just with him healing and his system being compromised… The lungs still healing, we just could not take that chance. I mean, we know when we leave them, you know, we are isolated still. Even when I go into work, I'm isolated. I stay away from people, you know.(Caregiver 0226314).

As a communicable disease, COVID‐19 affects households—particularly in this early phase of the epidemic before there was widespread vaccine availability, understanding of the role of ventilation, and ongoing mask and other personal protective equipment shortages. Consequently, one of the challenges for caregivers, who had often been sick themselves, was diminishing the risk of any additional infection and navigating receiving assistance while minimising personal contact with persons outside of the household. As one caregiver noted, “We didn't want anybody else to go through what he went through” (Caregiver 0101572). However, this additional isolation often increased familial care burden by reducing the level of community support they might receive. As another caregiver noted, “We still to this day, we still get offered support. And it's just because of social distancing and being safe is why we've had to more or less turn people down” (Caregiver 0226314). The *precautions* code was applied to highlight dialogue where the caregiver or patient altered their usual behaviour in order to prevent the spread of COVID‐19 to, or from, their household. In addition to highlighting the specific changes that caregivers and patients had to make in response to the pandemic, this theme provided insight into the extent of caution families took to avoid contracting or spreading the SARS‐CoV‐2 virus further.

#### 4.1.5 Addressing patient Independence

And then you get into, you know, arguments, but it's like you are constantly getting told what to do. Remind to take your meds. Did you do this, you do that. It's like…All right, now I feel like I'm being babied, you know, and not trusted.(Patient 1226314)

One of the more surprising findings of the study that became evident by interviewing dyads was how the need for, and assertion of, independence at times affected the familial relationship. Often caregivers expressed concerns around the safety and/or decision‐making capability of the patient who seemingly was doing more than perhaps they were ready to. As one caregiver noted, “I know he had some delirium, but he didn't want to acknowledge it” (Caregiver 0101572). This need of the caregiver to keep their loved one safe or to have necessary supports in place was often juxtaposed by the patients' desire to quickly return to their former level of independence and former familial role. As one patient aptly noted, “I don't wanna be a burden to anybody” (Patient 1101572). Despite the seriousness of an ICU hospitalisation and medically anticipated long post‐discharge recovery period, a common, and perhaps unrealistic, expectation of patients was a rapid return to their normal level of functioning. This theme was derived from the code *independence*.

#### 4.1.6 Engaging support services

There was a case manager and a social worker who were on the phone office. So nice. They said any time you have any questions, please call us. And they did. Yeah, they tried to help as much as they can.(Caregiver 0144604).

After the patient was discharged from the hospital setting, both members of the family dyad continued to need additional medical support for full recovery. The most frequently used homecare services by families included nursing support, occupational therapy and physical therapy. The additional medical support in the home allowed caregivers to feel cared for and, particularly in the case of therapy, provided patients with tangible hope of recovery.

They twice a week, they do leg exercises and stuff like that. To get my endurance back, you know, and I'm thankful for that, you know. They constantly did that and, as it went on, I got better…You know, that after hospital care was a great thing. You need it. You need it.(Patient 1174250)

Coming home following an ICU COVID‐19 discharge can be an emotional and challenging time for both patients and caregivers. The extra medical support in the immediate weeks following discharge played an important role in both caregiver and patient adjustment. However, at this early stage of the pandemic, several participants reported a significant delay in the initiation of home care services. Additionally, many caregivers noted that they declined services once they became available as they had become proficient in care delivery themselves. The theme of engaging support services was derived from the *ancillary support* code.

## 5 DISCUSSION

Collectively, all six themes (1) engaging the support of family and friends, (2) increased responsibilities to accommodate caregiving, (3) managing emotions, (4) managing infection control, (5) addressing patient independence and (6) engaging support services, provide a deeper understanding of the post‐discharge caregiving experiences, family support needs and points of intervention for clinicians. To the best of our knowledge, this is the first study to examine the adaptive needs of families following a COVID‐19 infection, or really any infective condition, using dyadic qualitative data. Though similar amounts of transcript data were provided for each dyad member, the increased prevalence of relevant quotes from caregivers is perhaps expected given the topical focus on family caregiving and patient expectations around of the temporariness of this role within this population. Further, though the families in this study were learning to adjust to the effects of a severe COVID‐19 infection that required intensive care treatment, the primary study findings, which describe the importance of social networks for family caregivers, the shifting of social roles and the need for emotional self‐regulation, have been similarly described in other populations.

For example, in a German longitudinal study of aging, Zwar et al. (2020) found that new family caregivers increased their social network size and had higher levels of depressive symptoms compared with non‐caregivers. Similarly, Roth (2020) evaluated data from an American longitudinal cohort study of older adults to identify the mechanism by which caregivers use social networks to increase access to support and resources. Specifically, they noted that connections to non‐related persons who do not know others in one's social network, or bridging ties, extends the potential capacity of the caregiver to gain access to novel information that can support care‐related tasks and allows for support without the same emotional attachment one would find in a bonding tie—generally a relative. Further, Roth found that caregivers were more likely to have bridging ties than non‐caregivers.

In our study, many families reported having various bridging ties—particularly fellow church members and, in some instances, neighbours. Our study finding that homecare and other ancillary support services were advantageous to families in adjusting to the caregiving role further supports the idea that interdisciplinary healthcare providers may also serve as bridging ties to families, thus extending their social support network. These additional types of bridging ties facilitated information gathering and a sense of well‐being. The increased likelihood of depressive symptoms in new family caregivers that was noted by Zwar et al. (2020) may explain the focus of our caregivers on emotional self‐regulation—and particularly remaining positive—a challenging feat for individuals adjusting to the traumatic experience of witnessing a loved one survive a life‐threatening illness, unexpectedly having to take on more responsibilities, and dealing with which shifting power dynamics within relationships. Czekanski (2017) noted that shifting role changes from relative (e.g., spouse or child) to caregiver is often complicated by the uncertainty of what the future might bring and may lead to feelings of grief and loss. Relatedly, Fox (2022), who describes their mental illness recovery journey within an autoethnography, discusses the identity crises that occurs as the patient/caregiver dyad negotiates and renegotiates their public (caregiving/receiving) relationship and private (familial) one. Inattention to the changing needs of either party can affect both individual and family unit functioning. It is clear that a primary challenge for families new to caregiving relationships is the adaptation to a new social role. This adaptation is perhaps complicated by the need to maintain infection control due to COVID‐19 and the assertion of independence by patients desiring to maintain their role within the family structure as it were pre‐hospitalisation. These changing familial relationships and related stress responses that are consequential to a loved one's COVID‐19 ICU‐related hospitalisation and discharge are consistent with the stress and coping relationships articulated in the Roy Adaptation Model (Roy, 2008). The Roy model may be used to guide the appropriate assessment of families and the development of effective interventions.

### 5.1 Adaptive coping and the Roy adaptation model

The Roy Adaptation Model (RAM) (Roy, 2008) is a stress and coping model that is used to guide nursing practice. As a primary goal of this model is to “enhance life processes to promote adaptation” (Roy, 2008, p. 28), the model is an optimal fit for understanding how pre‐ and post‐ICU discharge processes may be enhanced to improve family outcomes—particularly adaptation to caregiving. In addition to facilitating the connection of study findings to nursing practice, the model supports the advancement of nursing science by connecting and explaining the phenomena of adaptation as it relates to central metaparadigm concepts in nursing—persons, environment and health.

**FIGURE 1:**
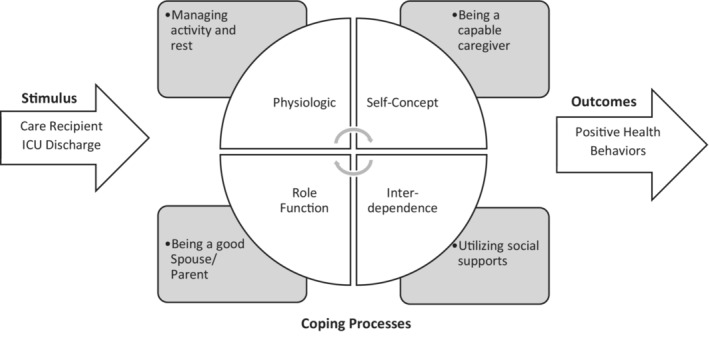
Family caregiving within the Roy adaptation model

The model is based on the premise that humans are holistic adaptive systems in constant interaction with their internal and external environments. To maintain optimal system functionality (i.e., health), humans must effectively cope with stress caused by various stimuli from their environments, such as an acute life‐threatening illness requiring caregiver support. The model posits that individuals and groups respond to stimuli through various coping processes that may be categorised as being in particular adaptive modes: physiologic (i.e., affecting the body), interdependence (i.e., reliance upon others), role function (i.e., who one is in relation to others) and self‐concept (i.e., beliefs about oneself) (See Figure 1). Each mode represents a type of challenge that the family, and particularly the caregiver, must deal with in order to adapt to the changes caused by the stress of providing or receiving care. Effective adaptation is evidenced by positive health behaviours. The healthcare team can develop effective interventions for ICU patients being discharged with COVID‐19 and their family caregivers by targeting specific adaptive modes or touch points of intervention.

#### 5.1.1 The physiologic mode

For example, in the physiologic mode, the primary adaptive concern is the need to meet the basic physical requirements of the body, particularly the need for activity and rest. Family caregivers increased responsibilities to accommodate caregiving likely leads to less rest, and more undesirable activities than expected, while recently discharged COVID‐19 patients likely need more rest. Some family caregivers in this study discussed significant physical challenges of providing care assistance with activities of daily living such as dressing, bathing and ambulation, while patients spoke about their desire to remain active and non‐burdensome. An effective pre‐hospital discharge intervention for families might include providing tips on getting adequate rest and the development of a plan for activity prioritisation and maintenance.

#### 5.1.2 The self‐concept mode

In the self‐concept mode, the primary adaptive need is for psychic and spiritual integrity or having confidence in oneself and who one is in order to exist with a positive self‐perception. Roy (2008) notes that often the sense of self is influenced by social interactions and perceptions about how one ought to be. For family caregivers, the idea of being a capable caregiver and what that means to them, likely influences their self‐concept. The fact that spirituality/spiritual support was a somewhat common theme amongst caregivers (*n* = 9) but not amongst patients (*n* = 1), is perhaps evidence of the challenge within this adaptive mode for caregivers. Clinicians may better assess for difficulties caregivers may be experiencing in coping with self‐concept by discussing with families what being a caregiver means and/or looks like to them, assisting with the development of realistic goals, and implementing necessary supports, such as training in aspects of care delivery, so that expectations may be more readily met.

#### 5.1.3 Role function

In the role function mode, adequate coping with challenges to the societal role an individual holds is necessary. The difficulties of this mode were perhaps best highlighted in how patients spoke about the need for independence and the challenge that this presented for family caregivers. Roy (2008) describes two particular concerns that evidence ineffective adaptation to changes in role function—role conflict and role failure. In role conflict there are inconsistent expectations around a particular role that are the result of conflict between roles, and in role failure, there is a failure to meet role expectations.

Family caregivers may experience conflict between their roles as family members and caregivers. Role conflicts may become particularly pronounced when the patient has a cognitive impairment, as is common following a COVID‐19 ICU discharge, and the caregiver must act in ways not congruent with the established and expected roles within that relationship. Further, caregiving needs may take precedence within the relationship and disallow optimally fulfilling the family role. There is a clear relationship between role function and self‐concept in that self‐concept affects role development. How a person perceives their ideal self will influence the expectations they set for themselves in role function. Assisting families with establishing adequate support systems that relieve caregiving responsibilities allows more energy to be devoted to familial roles and promotes patient autonomy.

#### 5.1.4 The interdependence mode

The final mode, interdependence, is characterised by the achievement of relational integrity or feeling secure in relationships and having a sense of being respected, loved and valued. This mode may be one of the most compromised adaptive modes for families adjusting to a recent COVID‐19‐related ICU discharge as primary relationships with significant others and support systems are affected. The compromised medical and psychological state of the patient may limit their ability to connect with the caregiver in the way that caregiver has been accustomed to. Similarly, the caregiver's shift from relative to caretaker can change how they engage with and are perceived by their loved one. Inevitably, these relationship changes may lead to loneliness in either, or both, dyad members and a sense of disconnection. Further, support systems, which would include any groups, organisations or people that help a person to achieve some purpose may be inadequate.

It is not typical for families to be aware of their support needs following a patient hospital discharge—particularly from the ICU (Picardi et al., 2021). Our findings underline the need for increased pre‐ and post‐discharge planning with an emphasis on social support and referrals. Necessary family adaptations that bolster family caregiving following a COVID‐19‐related ICU stay may be effectively facilitated by nurses. The Roy adaptation model provides an effective guide for assessing family adaptation to caregiving and identifying relevant and timely interventions. In addition to preparing families for the essential and realistic adjustments, they will need to make to transition towards recovery, or potentially a new normal, clinicians can be an important connection in family caregiving networks. Not only can clinicians provide useful support such as connecting caregivers with home health care and other available community‐based services, but they can also assist with immediate needs such as setting up follow‐up appointments and transportation if needed—tasks that reduce caregiver burden in the immediate weeks following discharge. It is also critical that clinicians help families to identify their established support systems before the patient discharges. Helping families to establish appropriate social supports pre‐discharge not only facilitates adaptation, but also reduces the likelihood of care burden, loneliness, isolation and poor caregiver/patient health outcomes.

### 5.2 Limitations

A few limitations of the study should be acknowledged. First, there was not a highly diverse sample of family dyads included in the study. Most participants were White and college‐educated with incomes over $50,000. Therefore, findings cannot be generalised to populations of minoritised and/or lower‐resourced populations. However, as with all qualitative studies that aim to understand human phenomena, such as familial adaptation following a critical care hospitalisation, examining situational experiences allows for the illumination of meanings, values and relationships that shed light on the human experience (Parse, 2001). Findings from this study may still be used to understand the pressing concerns of families, particularly the concerns of family caregivers. Second, the data analysed for this study were collected using interview scripts aimed at simply understanding the experiences of family dyads with COVID‐19 from initial illness thru discharge. As such, participant interviews may have lacked more in‐depth information about the specific strategies used by patients and caregivers to adapt to the family caregiving role. A future prospective study should consider focusing on recruiting more diverse samples and implementing interview designs that use open‐ended questions based on study objectives. In addition, examining the role of contextual factors such as income, age, religion and cultural identity on social networks and support may provide important insights on variances in family health outcomes amongst families who are adjusting to integrating a family caregiver.

## 6 CONCLUSION

Adequate adaptation to family caregiving of patients who have been recently discharged from the ICU with COVID‐19 requires compensatory changes in familial self‐care activities—namely prioritisation of caregiver personal health and wellness, along with attention to pre‐illness relationships. Without additional external support, either formally from clinicians or informally from social networks, relatives may become overwhelmed with the stress of family caregiving which may lead to negative health outcomes in both patients and caregivers. Clinicians, particularly nurses, play a critical role in helping to ensure that families have the support that they need before they leave the hospital. When social support is adequate, family caregiver/patient dyads can spend more time relating to one another as a family rather than just caregivers and patients. Ultimately, this shift in approach to focus on the needs of the family rather than just the needs of the patient facilitates family adaptation following a COVID‐19 ICU‐related hospitalisation.

## 7 RELEVANCE TO CLINICAL PRACTICE

Nurses play a critical role in the coordination of care and discharge planning processes. Assessing the family as the unit of care before discharge allows clinicians the opportunity to examine caregiver adaptive needs and facilitate timely connections to relevant supports. In particular, strategies to reduce challenges with the transition home such as caregiver skill building sessions initiated during hospitalisation and continued post‐hospitalisation may be promising in helping caregivers feel supported.

## FUNDING INFORMATION

This work was supported by the National Institute on Aging [P30AG053760 to S.G.R. and A.N.L., K01AG065420 to S.G.R and K01AG056557 to A.N.L.], the National Institute of Nursing Research [NR016914 to F.U.J.], Veterans Affairs Health Services Research and Development [IIR17‐045 and CR19 279 to T.J.I.], the University of Michigan Office of Research and the Procter & Gamble Company. Proctor & Gamble have not had any influence on the collection, management, analysis or interpretation of the data; preparation, review, or approval of the manuscript; or decision to submit the manuscript for publication. The content is solely the responsibility of the authors and does not necessarily represent the official views of the National Institutes of Health, the U.S. Government or Department of Veterans Affairs.

## CONFLICT OF INTEREST

The authors have no conflict of interest to report.

## Supporting information


**Appendix S1:** Supporting Information

## Data Availability

The data used in this study are available upon reasonable request from co‐author, Richard Gonzalez (gonzo@umich.edu). A rigorous curation process is presently underway to protect patient and caregiver confidentiality, after which the data will become publicly available in a secure data repository maintained by the Inter‐university Consortium for Political Research (https://www.icpsr.umich.edu/).
